# Relationship between volume status and possibility of pulmonary hypertension in dialysis naive CKD5 patients

**DOI:** 10.1371/journal.pone.0221970

**Published:** 2019-09-03

**Authors:** Byoung-Geun Han, Juwon Kim, In Young Jung, Jung-Woo Son

**Affiliations:** 1 Department of Nephrology, Yonsei University Wonju College of Medicine, Wonju, Kang-won, Korea; 2 Department of Laboratory Medicine, Yonsei University Wonju College of Medicine, Wonju, Kang-won, Korea; 3 Department of Internal Medicine, Yonsei University Wonju College of Medicine, Wonju, Kang-won, Korea; 4 Department of Cardiology, Yonsei University Wonju College of Medicine, Wonju, Kang-won, Korea; University of Liège, BELGIUM

## Abstract

**Background:**

Chronic fluid overload is common in patients with chronic kidney disease (CKD) and can with time lead to poor prognosis regarding to the cardiovascular events. Serum NT-proBNP and OH/ECW might reflect fluid status of the patients, and the maximal tricuspid regurgitation velocity (TRVmax) could reflect systolic pulmonary artery pressure (SPAP). We investigated the relationship between markers of volume status and marker of pulmonary hypertension (PH) in non-dialysis CKD5 (CKD5-ND) patients.

**Methods:**

Bioimpedance spectroscopy (BIS), echocardiography, and measurement of serum NT-proBNP were performed in 137 consecutive patients on the same day. TRVmax greater than or equal to 2.9 m/s, corresponding to SPAP of approximately 36 mmHg, was used as a definition of the possibility of PH in the absence of left heart disease and chronic respiratory disease (PH group).

**Results:**

Patients with possibility of PH (TRVmax ≥ 2.9 m/s) was found in 27 (19.70%) patients. Among the values obtained from BIS, those reflecting the fluid balance (OH, OH/ECW, and E/I ratio) were significantly higher in the PH group. The OH/ECW in patients with PH were significantly higher than those patients without (26.76 ± 15.07 vs. 13.09 ± 15.05, P < 0.001). NT-proBNP was also significantly higher in PH group compared to the non-PH group (median = 10,112 pg/ml, IQR = 30,847 pg/ml vs. median = 1,973 pg/ml, IQR = 7,093 pg/ml, P < 0.001). OH/ECW was positively associated with TRVmax (r = 0.235, P = 0.006). Multivariate logistic regression revealed that increased OH/ECW and serum NT-proBNP were significantly associated with an increased risk of PH.

**Conclusions:**

A significant number of patients showed increased TRVmax, which was closely related to volume status in CKD5-ND patients. Echocardiography and BIS could be important players in a high possibility of PH detection and treatment in asymptomatic CKD patients. Therefore, these measures could be helpful to improve the cardiac outcomes after initiating renal replacement therapy. Further research may be needed to validate the consistency of this association across other stages of CKD.

## Introduction

Pulmonary hypertension (PH) is a disease that can progress to right heart failure, whatever the cause, and associated with a poor prognosis [1]. Recently, it has been recognized as a novel threat to end stage renal disease (ESRD) patients and has become a subject of much interest. However, its pathogenesis has not been completely elucidated. The reported prevalence of PH in chronic kidney disease (CKD) patients, mostly undergoing hemodialysis, widely varied and increased with the decline of renal function [2]. The rate of PH was 33.7% - 52% in hemodialysis patients [3–6] and 12.6% - 42% in peritoneal dialysis patients [7–9]. However, there has not been many studies conducted with CKD patients without dialysis treatment, and the reported range of prevalence was 8.3% -71% [2, 4, 10–15]. The prevalence of PH increased with longer duration of dialysis treatment [10].

The prevalence of PH varies between reports due to the lack of consideration of fluid overload and the stage of CKD [16]. Fluid overload represents a crucial step in the pathophysiological pathways to PH in CKD patients. Assessment of volume status in patients with renal insufficiency is important not only for short-term volume management but also for long-term prevention of cardiovascular disease. Fluid overload is one of the predictors of mortality and morbidity. However, the traditional methods have several limitations for detecting the degree and severity of overhydration [17]. Recently, bioimpedance spectroscopy (BIS) performed at the bedside has gained popularity because it is a non-invasive, quick and relatively affordable method to quantitatively assess the volume status of the patient, although only a small number of studies have examined the relationship between volume status and PH [6, 8, 18, 19]. We previously reported that increased E/e´ ratio, reflecting left ventricular diastolic dysfunction (LVDD), was strongly associated with fluid overload in CKD patients [20]. LVDD may contribute to the development of PH by causing an elevated left atrial pressure [14].

Herein, we particularly focused on the relationship between volume status, estimated by multi-frequency bioimpedance and NT-proBNP, and PH in dialysis naive CKD5 patients.

## Materials and methods

### Patients and data collection

This study was initiated after receiving approval from the Institutional Review Board of Yonsei University Wonju Severance Christian Hospital (institutional review board no. CR 318087) and enrolled only the patients who provided written informed consent prior to the study. This study is an observational cross-sectional and prospective study of patients hospitalized for a renal replacement therapy plan. BIS, echocardiography, and laboratory tests were performed at the same time before the first dialysis, either hemodialysis or peritoneal dialysis. Patients with liver cirrhosis (n = 1), infection (n = 1), atrial fibrillation (n = 2), and valvular heart disease (n = 1) were excluded from the analysis. Patients (n = 8) with left ventricular ejection fraction (LVEF) less than 45% were also excluded. None of the patients had chronic respiratory disease. 137 patients were finally included in the analysis. This study was conducted in accordance with the Declaration of Helsinki.

### Echocardiographic assessment

Echocardiography was performed in the harmonic imaging mode using a 3-MHz transducer and commercial ultrasound system (Vivid-7; General Electric-Vingmed, Milwaukee, WI, USA). A TRV was recorded from the parasternal or apical window with the continuous wave Doppler probe. The left atrial (LA) dimension was measured by 2D-guided M-mode echocardiography using the parasternal short-axis view at the base of the heart. Three LA dimensions were used to calculate the LA volume as an ellipse using the formula: LA volume = (π/6)(SA1×SA2×LA), where SA1 is the M-mode LA dimension, and SA2 and LA are measurements of the short- and long-axis with the apical four-chamber view at ventricular end-systole, respectively. The LA volume index (LAVI) was calculated by dividing LA volume by body surface area (BSA) using the formula as follows: BSA = 0.007184 × weight^0.425^× height^0.725^ (m^2^). LV internal dimensions, LV wall thickness, and LVEF, measured using the biplane modified Simpson rule, according to the previously reported recommendations [21]. LV mass was calculated following American Society of Echocardiography recommendations as LV mass (g) = 1.04×([PWTd+SWTd+LVDd]^3^×[LVEDD]^3^)×0.8+0.6, where PWTd and SWTd are the posterior and septal wall thickness at end-diastole respectively, and LVEDD is the M-mode LV dimension with the short axis view at end-diastole. To correct for body surface area, the LV mass index (LVMI) was calculated as LV mass/BSA. As recommended by the Heart Failure and Echocardiography Associations of European Society of Cardiology, both conventional and tissue Doppler imaging (TDI) echocardiographic techniques were used for the evaluation of LV diastolic function [22].

The maximal TRV (TRVmax) greater than or equal to 2.9 m/s, corresponding to systolic pulmonary artery pressure (SPAP) of approximately 36 mmHg, was used as a definition of a high possibility of PH (PH group) [23]. Echocardiography was performed by trained cardiologists who were completely blinded to the patient information. The intra-class correlation coefficient of intra- and inter-observer variability in measurement of TRVmax was 98.9% and 98.0%.

### Assessment of the volume status

Bioimpedance spectroscopy using the BCM^TM^ (Body Composition Monitoring^TM^, Fresenius Medical Care, Bad Homburg, Germany) was performed prior to any dialysis treatment. BCM^TM^ utilizes alternating electric currents across 50 different frequencies from 5 to 1000 kHz for the measurement of fluid status. Extracellular water (ECW), intracellular water (ICW) and total body water (TBW) were automatically calculated. The overhydration (OH) value can be calculated from the difference between the normal ECW and actual measured ECW [24]. OH/ECW greater than or equal to 15% was defined as fluid overload [20]. Patients were in supine position. Disposable electrode patches were used for all measurements.

### Laboratory evaluations

All laboratory studies were performed before the first dialysis application. NT-proBNP, which is known to reflect volume status, was also measured, and the association with the possibility of PH was analysed. NT-proBNP was measured using electrochemiluminescence immunoassay (ECLIA) on Modular analytics E170 (Roche Diagnostics, Mannheim, Germany). The analytical measurement range for NT-proBNP was 5 to 35,000 pg/mL. In the correlation analysis, it was not appropriate to use the NT-proBNP value because accurate values of less than 5 pg/mL or greater than 35,000 pg/mL could not be obtained. Therefore, patients were divided into three groups in accordance with tertiles of NT-proBNP for the analysis.

### Statistical analysis

All analyses were performed with IBM Statistics Package for the Social Science version 23.0 (IBM Corporation, Armonk, NY, USA). The study population characteristics are presented as the mean ± SD, median (IQR), or total number (percentage). Based on the results of TRVmax, the patients were categorized into two groups according to the presence of the possibility of PH. Differences in clinical variables between the two groups were tested with two-sample t-test for continuous variables. The nominal variables were compared using Chi-square test or Fisher’s exact test as appropriate. Pearson’s correlation analysis was used to examine correlations between TRVmax and other variables such as volume markers (OH/ECW, NT-proBNP) and echocardiographic variables. Multivariate logistic regression models was used to examine the associations of OH/ECW and NT-proBNP with TRVmax. The C-statistics was utilized to test the predictive accuracy of a logistic regression model. Goodness of fit for model was assessed using the Hosmer-Lemeshow test, whereby we considered a value of P < 0.05 to indicate that the model had a poor fit. In this analysis, we adjusted for age and sex (model 1) and for diuretics use and albumin (model 2). Finally, a receiver operating characteristic (ROC) curve was created to establish cut-off values of OH and OH/ECW that discriminate patients with TRVmax ≥ 2.9 m/s from those with TRVmax < 2.9 m/s. Statistical significance was defined as P < 0.05.

## Results

### Patients’ characteristics

The characteristics of the study population are shown in Table 1. Patients with the possibility of PH (TRVmax ≥ 2.9 m/s) were less commonly treated with diuretics (P = 0.006). PH group had significantly lower estimated glomerular filtration rate. The age and gender were similar. The mean age of male and female patients was 59.99 ± 13.09 years and 59.90 ± 11.80 years, respectively. The median [interquartile range (IQR)] OH and OH/ECW were 2.0 (0.5–5.2) L and 13.48 (4.11–27.70) %, respectively. The median NT-proBNP was 2,570.0 (759.2–9,200.5) pg/ml.

**Table 1:** Demographic findings of study patients.

Variables	Total (N = 137)	TRVmax		P-value
		< 2.9 m/s(N = 110)	≥ 2.9 m/s (N = 27)	
Age, years	59.95±12.52	66.75±12.40	56.70±12.73	0.133
Sex Men	80 (58.4%)	65 (59.1%)	15 (55.6%)	0.738
Women	57 (41.6%)	45 (40.1%)	12 (44.4%)	
BMI (kg/m^2^)	24.51±3.98	24.26±3.84	25.56±4.45	0.128
Diabetes Yes	79 (57.7%)	59 (53.6%)	20 (74.1%)	0.054
Non diabetic renal disease	58 (42.3%)	51 (46.4%)	7 (25.9%)	
Hypertension	13	11	2	
CGN	13	12	1	
FSGS	4	4	-	
IgAN	7	4	3	
MGN	1	1	-	
MPGN	1	-	1	
Hereditary/congenital disease	6	6	-	
Other	4	4	-	
Unknown	9	9	-	
BP medication Yes	129 (94.2%)	102 (92.7%)	27 (100.0%)	0.355
No	8 (5.8%)	8 (7.3%)	0 (0.0%)	
Diuretics Yes	91 (66.4%)	67 (60.9%)	24 (88.9%)	0.006
No	46 (33.6%)	43 (39.1%)	3 (11.1%)	
SBP (mmHg)	143.73±19.42	143.73±19.42	143.73±19.42	0.090
DBP (mmHg)	79.60±11.62	79.60±11.62	79.60±11.62	0.609
eGFR (mL/min/1.73 m^2^)	6.87±2.63	7.09±2.77	6.02±1.72	0.014

CGN: chronic glomerulonephritis; DBP: diastolic blood pressure; eGFR: estimated glomerular filtration rate; FSGS: focal segmental glomerulosclerosis; IgAN: immunoglobulin A nephropathy; MGN: membranous glomerulonephritis; MPGN: membranoproliferative glomerulonephritis; SBP: systolic blood pressure; TRVmax: maximal tricuspid regurgitation velocity.

### Differences according to *TRVmax*

Among the BIS parameters, extracellular water/intracellular water ratio (E/I ratio), ECW, OH, and OH/ECW were significantly greater in PH group. Serum parameters were not different between the two groups except iPTH and NT-proBNP (Table 2). NT-proBNP was significantly higher in patients with the possibility of PH compared to those without PH. Among the echocardiographic parameters, the LA dimension, LAVI, E velocity, and E/e´ ratio were significantly greater in PH group, while the A velocity, e´ velocity, and LVEF were not significantly different.

**Table 2:** Compared parameters (laboratory, bioimpedence and echocardiography) according to binary TRVmax.

variables	Total (N = 137)	TRVmax		P-value
		< 2.9 m/s (N = 110)	≥ 2.9 m/s (N = 27)	
OH (liter)	3.26±4.11	2.63±3.91	5.84±3.94	< 0.001
OH/ECW (%)	15.75±15.96	13.09±15.05	26.76±15.07	< 0.001
E/I ratio	1.01±0.22	0.98±0.20	1.14±0.23	< 0.001
ECW (liter)	16.98±5.06	16.24±4.75	19.98±5.26	< 0.001
ICW (liter)	16.90±3.97	16.70±3.88	17.74±4.28	0.221
TBW (liter)	33.80±8.17	32.94±7.76	37.33±8.99	0.012
LA dimension (cm)	4.70±0.48	4.63±0.44	4.99±0.52	< 0.001
LAVI (ml/m^2^)	38.90±11.12	36.21±9.06	49.85±12.13	< 0.001
E (m/s)	0.84±0.31	0.75±0.24	1.20±0.31	< 0.001
A (m/s)	1.00±0.29	1.00±0.22	1.00±0.48	0.943
e´ (m/s)	0.06±0.02	0.05±0.02	0.06±0.02	0.078
E/e´ ratio	15.85±5.65	14.58±4.20	20.96±7.66	< 0.001
LVEDD (cm)	5.42±0.53	5.36±0.53	5.66±0.47	0.008
LVEDV (ml)	144.99±31.10	141.59±30.54	158.85±30.01	0.009
LVMI (g/m^2^)	118.78±30.84	115.34±30.94	132.82±26.60	0.008
LVEF (%)	63.95±5.93	63.99±5.82	63.78±6.46	0.868
NT-proBNP, (pg/mL) *	2,570 (759–9,201)	1,973 (592–7,686)	10,112 (4,153–35,000)	< 0.001
Hemoglobin (g/dL)	9.10±1.31	9.18±1.37	8.77±0.99	0.143
Protein (g/dL)	6.16±0.77	6.21±0.80	5.96±0.61	0.126
Albumin (g/dL)	3.47±0.58	3.51±0.60	3.34±0.47	0.178
Cholesterol (mg/dL)	151.90±48.71	151.00±50.20	158.00±42.30	0.489
Triglyceride (mg/dL)	123.60±73.32	125.70±74.55	115.20±68.79	0.507
HDL (mg/dL)	40.00±14.31	39.23±14.05	43.24±15.23	0.209
LDL (mg/dL)	87.27±42.43	86.38±40.02	91.00±35.50	0.627
iPTH (pg/mL)	308.06±206.84	319.26±225.25	260.65±83.20	0.032
Calcium (mg/dL)	7.66±1.10	7.74±1.14	7.30±0.81	0.062
Phosphorus (mg/dL)	5.96±1.60	5.93±1.60	6.09±1.61	0.655
Uric acid (mg/dL)	8.10±2.46	8.10±2.39	8.10±2.79	1.000
Magnesium (mg/dL)	2.32±0.48	2.28±0.48	2.47±0.48	0.079
TSAT (%)	30.96±16.42	32.33±17.54	25.35±8.93	0.067

* Median (Interquartile range)

ECW: extracellular water; E/I: extracellular water/ intracellular water; HDL: High-density lipoprotein; iPTH: intact parathyroid hormone; ICW: intracellular water; LA: left atrium; LAVI: left atrium volume index; LDL: low-density lipoprotein; LVEDD: left ventricular end-diastolic dimension; LVEDV: left ventricular end-diastolic volume; LVEF: left ventricular ejection fraction; LVMI: left ventricular mass index; NT-proBNP: N-terminal pro-hormone of brain natriuretic peptide; OH: overhydration; TBW: total body water; TRVmax: maximal tricuspid regurgitation velocity; TSAT: transferrin saturation.

### Correlation between laboratory, echocardiographic parameters and markers of volume status

TRVmax was correlated with volume marker (OH, OH/ECW, E/I ratio). OH/ECW was significantly associated with LAVI, E velocity, E/e´ ratio, LVEDD, and LVEDV (Table 3).

**Table 3:** Correlation between laboratory, echocardiographic parameters and markers of volume status.

	TRVmax		OH/ECW	
Variables	Corr. coeff	P-value	Corr. coeff	P-value
SBP (mmHg)	0.115	0.182	0.235	0.006
DBP (mmHg)	-0.042	0.629	0.189	0.027
Total Protein (g/dL)	-0.137	0.110	-0.510	< 0.001
Albumin (g/dL)	-0.141	0.101	-0.551	< 0.001
iPTH (pg/mL)	-0.163	0.057	-0.253	0.003
OH (liter)	0.189	0.027	0.920	< 0.001
OH/ECW (%)	0.235	0.006	-	-
E/I ratio	0.185	0.031	0.861	< 0.001
ECW (liter)	0.165	0.055	0.733	< 0.001
ICW (liter)	0.059	0.495	0.075	0.383
TBW (liter)	0.123	0.151	0.492	< 0.001
LA dimension (cm)	0.300	< 0.001	0.253	0.003
LAVI (ml/m^2^)	0.450	< 0.001	0.368	< 0.001
E (m/s)	0.483	< 0.001	0.461	< 0.001
A (m/s)	-0.107	0.216	0.049	0.571
e´ (m/s)	0.174	0.043	0.167	0.052
E/e´ ratio	0.329	< 0.001	0.322	< 0.001
LVEDD (cm)	0.172	0.044	0.228	0.007
LVEDV (ml)	0.185	0.030	0.242	0.004
LVMI (g/m^2^)	0.223	0.009	0.157	0.066
LVEF (%)	0.153	0.074	-0.094	0.274
TRVmax (m/s)	-	-	0.235	0.006

Corr. coeff., correlation coefficient

ECW: extracellular water; E/I: extracellular water/ intracellular water; DBP: diastolic blood pressure; iPTH: intact parathyroid hormone; ICW: intracellular water; LA: left atrium; LAVI: left atrium volume index; LVEDD: left ventricular end-diastolic dimension; LVEDV: left ventricular end-diastolic volume; LVEF: left ventricular ejection fraction; LVMI: left ventricular mass index; OH: overhydration; SBP: systolic blood pressure; TBW: total body water; TRVmax: maximal tricuspid regurgitation velocity.

### Multivariate analysis using logistic regression

Among the significantly different variables between the two groups, OH/ECW and NT-proBNP were associated with TRVmax in unadjusted model. These variables were chosen considering the collinearity among the factors. OH/ECW was divided into two groups: OH/ECW <15%, and OH/ECW ≥15% (fluid overload). We also categorized NT-proBNP as tertiles (<1,204.62 pg/mL, 1,204.62~6,931.55 pg/mL, >6,931.55 pg/mL). Odds ratio was calculated using the lowest tertile as the reference.

The fluid overloaded group showed significant odds ratio in model 2 whereas the highest NT-proBNP group showed significant odds ratio in model 1 and model 2 (Table 4). The Hosmer–Lemeshow tests showed significant goodness of fit for model 1 (P = 0.061) and model 2 (P = 0.958). Model 2 showed high capacity for predicting the high risk of PH group. The c-statistic was 0.769 (95% CI 0.676 ~0.861, P < 0.001) and 0.788 (95% CI 0.695 ~0.880, P < 0.001) for model 1 and model 2, respectively.

**Table 4:** Multivariate analysis using logistic regression: Predictive factors for the risk of pulmonary hypertension group.

variables	Univariate analysis		Model 1		Model 2	
	OR (95%CI)	P-value	OR (95%CI)	P-value	OR (95%CI)	P-value
OH/ECW						
<15 (%)	Reference		Reference		Reference	
≥15 (%)	5.453 (2.037~14.601)	< 0.001	2.860 (0.920~8.889)	0.069	3.463 (2.037~14.601)	0.049
NT-proBNP						
<1,204 (pg/mL)	Reference		Reference		Reference	
1,204~ 6,931 (pg/mL)	5.230 (1.062~25.747)	0.042	3.931 (0.729~21.211)	0.111	4.530 (0.805~25.495)	0.087
> 6,931 (pg/mL)	11.862 (2.534~55.530)	0.002	6.055 (1.113~32.949)	0.037	6.184 (1.094~34.956)	0.039

Model 1: Adjusted for age and sex.

Model 2: Adjusted for age, sex, diuretics use, and albumin.

CI, confidence interval; OR, odds ratio

ECW: extracellular water; NT-proBNP: N-terminal pro-hormone of brain natriuretic peptide; OH: overhydration.

## Discussion

The pathogenesis of PH in ESRD patients is still complex and not completely understood. Several factors, such as fluid overload, arteriovenous fistula, anemia, hypoalbuminemia, cardiac dysfunction, mineral bone disease (MBD), uremic vasculopathy, and non-biocompatible dialysis membranes, have been proposed for the development of PH in dialysis patients [8, 25–27]. Different from other previous reports, albumin and hemoglobin levels and markers of CKD-MBD were not associated with the possibility of PH in this study [9, 28]. In addition, because the patients were dialysis naïve CKD, the arteriovenous fistulae and dialysis membrane components, which were associated with PH in other studies, were excluded in this study [14, 15, 29, 30].

Serum NT-proBNP levels is a biochemical marker for estimating the volume status and is associated with LV diastolic dysfunction [31]. In the present study, we found that E/e´ ratio, a marker of diastolic dysfunction, was associated with volume status and significantly correlated with NT-proBNP and TRVmax. Tissue Doppler e´ velocity, which is an element of E/e´ ratio, was related to TRVmax but not to OH/ECW and NT-proBNP. E velocity was associated with TRVmax, OH/ECW, and NT-proBNP, and there was a significant difference between the patients with or without PH, whereas e´ velocity was not. These findings are in line with the study that E velocity is associated with the circulating volume of hemodialysis patients [32]. On the other hand, A velocity was not related to all three factors. Diastolic dysfunction may contribute to the development of PH by causing an elevated left atrial pressure [12, 14, 23]. Progressive fluid overload with diastolic dysfunction could increase pulmonary capillary wedge pressure, which may result in PH. The right ventricle with thin muscle equipment usually operates at low blood pressure and is not able to tolerate high vascular resistance. Once the right heart chambers lose their distensibility due to an elevated left atrial pressure, it leads to tricuspid regurgitation and further right heart volume overload [33]. PH might be induced and/or aggravated by left heart disease. Because we excluded patients with systolic dysfunction, the main factor would probably be the diastolic dysfunction. Therefore, volume overload is critical to determining TRVmax.

Since the symptoms and signs of PH are somewhat ambiguous, nephrologists rarely suspect PH at the beginning of treatment in the patients with CKD [34]. Clinically, an indication of diuretic therapy is given when the patient complains of the signs and symptoms of fluid overload. It is not common clinical practice to prophylactically administer diuretics prior to diagnosis of PH. Nevertheless, in this study, diuretics were given to many PH patients (88.9%). However, as this study was conducted retrospectively, we could not confirm whether there was a need for the clinicians to prescribe diuretics in suspicion of PH. Furthermore, patients with PH demonstrated more frequent diabetes, and 95% (19 patients) of these patients were on diuretics. Endothelial dysfunction and increased arterial stiffness may occur in early diabetic CKD and so forth fluid overload may occur subsequently and have an effect on left ventricular systolic and/or diastolic dysfunction in late diabetic CKD. Therefore, the authors conclude that the presence of diabetes may have an effect on PH, but the numbers of cases included in this study are small so further studies and analysis with larger numbers of patients, divided into the early and late stage, is needed. In general, the diagnosis of PH can be made in collaboration with a cardiologist with the consideration of two things. The first is that the right heart catheterization should be performed by a skillful specialist because of the invasive nature of the procedure, and the need for a special space equipped with special instruments. The second issue is whether volume control can improve PH, because volume overload is a modifiable factor. However, when CKD5 patients initiate incident dialysis, a comprehensive approach should be implemented because there are factors that can induce PH other than fluid overload.

**Fig 1:**
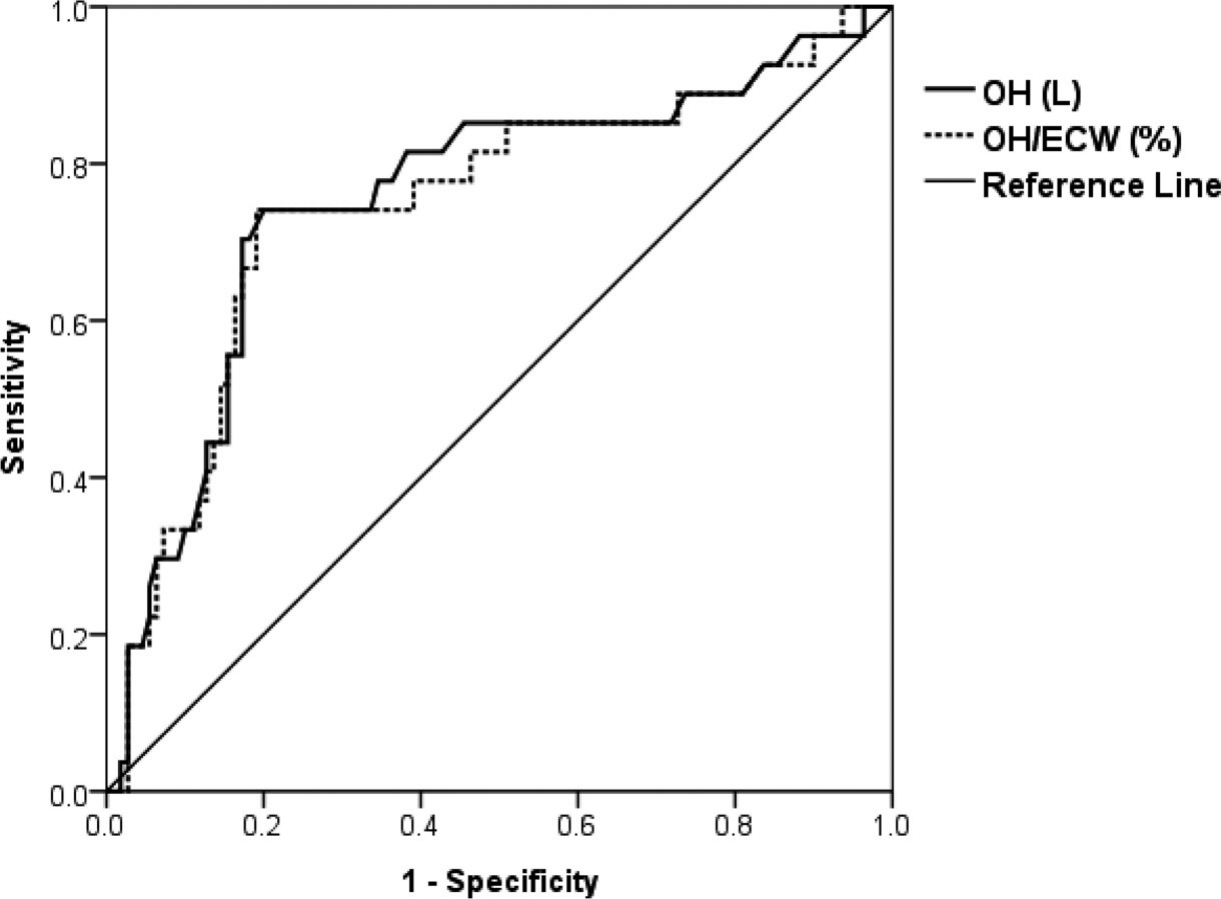
Receiver operating characteristics (ROC) curves of OH and OH/ECW for predicting the possibility of PH (TRVmax ≥ 2.9 m/s). Areas under the curve: OH 0. 754 (95% CI 0.643–0.866) and OH/ECW 0.746 (95% CI 0.633–0.860).

We could not confirm the change of SPAP by volume control after initiating dialysis. However, since volume control is a reversible factor, improvement of PH can be expected by correcting it. In fact, Yilmaz *et al*. reported that the SPAP, OH/ECW, and the frequency of PH were significantly reduced after hemodialysis treatment [6]. BIS can be useful as a tool for this, which enables treatments targeting not only PH but also LVDD [35]. An OH/ECW of 15% is thought to represent approximately 2.4 liters of overhydration and is used as a cut-off value for overhydration. In our previous report, the cut-off value for OH and OH/ECW predicting E/e´ ratio greater than 15 was 2.45 liters and 17.28%, respectively [31]. Furthermore, ROC curves were drawn for OH and OH/ECW to determine the cut-off values predicting TRVmax ≥ 2.9 m/s. The area under curve (AUC ± standard error) and cut-off value were as follows: 0.754 ± 0.057 (P < 0.001) and 4.1 liters (sensitivity 74.1%, specificity 80.0%) for OH; 0.746 ± 0.058 (P < 0.001) and 22.66% (sensitivity 74.1%, specificity 80.9%) for OH/ECW (Fig 1). If the fluid overload is above a certain level, it is better to recognize the need for echocardiography and to confirm the presence or absence of PH. Despite the correction of fluid overload with dialysis treatment, it is desirable to consider kidney transplantation rather than dialysis treatment in patients with irreversible PH [36, 37]. At this stage, the role of cardiologist in determining the mode of renal replacement therapy is crucial.

As a limitation of our study, this was a single-center study that included a relatively small number of patients. Furthermore, although SPAP may increase with age and in obesity, weight or body mass index was not adjusted for multivariate logistic regression models. Also, the lack of serial echocardiograms to assess changes in cardiac structure and function over time in relation to the volume control by renal replacement therapy, even though the definition of PH by echocardiography used in this study has limited sensitivity and specificity. Therefore, the mechanism by which fluid overload contributes to PH could not be proven in our study. Finally, although it could have given us important clues to our hypothesis, we did not perform the mandatory procedure, right heart catheterization, for diagnosis confirmation. Despite these limitations, the strength of this study is that we had objectively measured the volume status and concurrently have blindly evaluated SPAP. The study population was relatively homogenous and the patients were mostly enrolled to the study before making the arteriovenous fistula. As far as we know, this is the first study to have attempted in dialysis naïve CKD patients.

Taken together, a significant number of patients showed increased SPAP, which was closely related to volume status in CKD5-ND patients. Therefore, echocardiography and BIS could play an important role in PH detection and treatment of asymptomatic CKD patients. Our study suggests the need for calculation and follow-up of pulmonary artery pressure using Doppler echocardiography in all patients with established CKD5-ND before considering the dialysis treatment. If possible, it is advisable to perform the right heart catheterization in order to confirm PH. These process could be helpful to improve the cardiac outcomes after initiating renal replacement therapy.

## Supporting information

S1 File Dataset: Demographic, laboratory, bioimpedence, and echocardiographic data. (XLSX)

## Abbreviations

DBP: diastolic blood pressure
eGFR: estimated glomerular filtration rate
ECW: extracellular water
ICW: intracellular water
LAVI: left atrium volume index
LVEDD: left ventricular end-diastolic dimension
LVEDV: left ventricular end-diastolic volume
LVDD: left ventricular diastolic dysfunction
LVEF: left ventricular ejection fraction
LVMI: left ventricular mass index
NT-proBNP: N-terminal pro-hormone of brain natriuretic peptide
OH: overhydration
PH: pulmonary hypertension
SBP: systolic blood pressure
SPAP: systolic pulmonary artery pressure
TBW: otal body water
TRV: tricuspid regurgitation velocity

## References

[1] Tang M, Batty JA, Lin C, Fan X, Chan KE, Kalim S. Pulmonary hypertension, mortality, and cardiovascular disease in CKD and ESRD patients: A systematic review and meta-analysis. Am J Kidney Dis. 2018;72:75–83 10.1053/j.ajkd.2017.11.018 29429751

[2] Reque J, Garcia-Prieto A, Linares T, Vega A, Abad S, Panizo N, et al Pulmonary hypertension is associated with mortality and cardiovascular events in chronic kidney disease patients. Am J Nephrol. 2017;45:107–114 10.1159/000453047 27941345

[3] Agarwal R. Prevalence, determinants and prognosis of pulmonary hypertension among hemodialysis patients. Nephrol Dial Transplant. 2012;27:3908–3914 10.1093/ndt/gfr661 22290987 PMC3484729

[4] Yigla M, Nakhoul F, Sabag A, Tov N, Gorevich B, Abassi Z, et al Pulmonary hypertension in patients with end-stage renal disease. Chest. 2003;123:1577–1582 10.1378/chest.123.5.1577 12740276

[5] Mahdavi-Mazdeh M, Alijavad-Mousavi S, Yahyazadeh H, Azadi M, Yoosefnejad H, Ataiipoor Y. Pulmonary hypertension in hemodialysis patients. Saudi J Kidney Dis Transpl. 2008;19:189–193 18310865

[6] Yilmaz S, Yildirim Y, Taylan M, Demir M, Yilmaz Z, Kara AV, et al The relationship of fluid overload as assessed by bioelectrical impedance analysis with pulmonary arterial hypertension in hemodialysis patients. Med Sci Monit. 2016;22:488–494 10.12659/MSM.896305 26874785 PMC4755666

[7] Xu Q, Xiong L, Fan L, Xu F, Yang Y, Li H, et al Association of pulmonary hypertension with mortality in incident peritoneal dialysis patients. Perit Dial Int. 2015;35:537–544 10.3747/pdi.2013.00332 25185012 PMC4597986

[8] Unal A, Sipahioglu M, Oguz F, Kaya M, Kucuk H, Tokgoz B, et al Pulmonary hypertension in peritoneal dialysis patients: prevalence and risk factors. Perit Dial Int. 2009;29:191–198 19293357

[9] Kumbar L, Fein PA, Rafiq MA, Borawski C, Chattopadhyay J, Avram MM. Pulmonary hypertension in peritoneal dialysis patients. Adv Perit Dial. 2007;23:127–131 17886618

[10] Issa N, Krowka MJ, Griffin MD, Hickson LJ, Stegall MD, Cosio FG. Pulmonary hypertension is associated with reduced patient survival after kidney transplantation. Transplantation. 2008;86:1384–1388 10.1097/TP.0b013e318188d640 19034007

[11] Bolignano D, Rastelli S, Agarwal R, Fliser D, Massy Z, Ortiz A, et al Pulmonary Hypertension in CKD. Am J Kidney Dis. 2013;61:612–622 10.1053/j.ajkd.2012.07.029 23164943

[12] Pabst S, Hammerstingl C, Hundt F, Gerhardt T, Grohe C, Nickenig G, et al Pulmonary hypertension in patients with chronic kidney disease on dialysis and without dialysis: results of the PEPPER-study. PLoS One. 2012;7:e35310 10.1371/journal.pone.0035310 22530005 PMC3329424

[13] Yigla M, Fruchter O, Aharonson D, Yanay N, Reisner SA, Lewin M, et al Pulmonary hypertension is an independent predictor of mortality in hemodialysis patients. Kidney Int. 2009;75:969–975 10.1038/ki.2009.10 19212417

[14] Abdelwhab S, Elshinnawy S. Pulmonary hypertension in chronic renal failure patients. Am J Nephrol. 2008;28:990–997 10.1159/000146076 18635926

[15] Havlucu Y, Kursat S, Ekmekci C, Celik P, Serter S, Bayturan O, et al Pulmonary hypertension in patients with chronic renal failure. Respiration. 2007;74:503–510 10.1159/000102953 17505128

[16] Zhang Q, Wang L, Zeng H, Lv Y, Huang Y. Epidemiology and risk factors in CKD patients with pulmonary hypertension: a retrospective study. BMC Nephrol. 2018;19:70 10.1186/s12882-018-0866-9 29554879 PMC5859392

[17] Kraemer M, Rode C, Wizemann V. Detection limit of methods to assess fluid status changes in dialysis patients. Kidney Int. 2006;69:1609–1620 10.1038/sj.ki.5000286 16501488

[18] Yoo HHB, Dos Reis R, Telini WM, Telini LR, Hueb JC, Bazan SGZ, et al Association of pulmonary hypertension with inflammation and fluid overload in hemodialysis patients. Iran J Kidney Dis. 2017;11:303–308 28794293

[19] Yoo HHB, Martin LC, Kochi AC, Rodrigues-Telini LS, Barretti P, Caramori JT, et al Could albumin level explain the higher mortality in hemodialysis patients with pulmonary hypertension? BMC Nephrol. 2012;13:80 10.1186/1471-2369-13-80 22867112 PMC3489568

[20] Han BG, Song SH, Yoo JS, Park H, Kim J, Choi E. Association between OH/ECW and echocardiographic parameters in CKD5 patients not undergoing dialysis. PLoS One. 2018;13:e0195202 10.1371/journal.pone.0195202 29630661 PMC5891010

[21] Lang RM, Bierig M, Devereux RB, Flachskampf FA, Foster E, Pellikka PA, et al Recommendations for chamber quantification: a report from the American Society of Echocardiography's Guidelines and Standards Committee and the Chamber Quantification Writing Group, developed in conjunction with the European Association of Echocardiography, a branch of the European Society of Cardiology. J Am Soc Echocardiogr. 2005;18:1440–1463 10.1016/j.echo.2005.10.005 16376782

[22] Nagueh SF, Appleton CP, Gillebert TC, Marino PN, Oh JK, Smiseth OA, et al Recommendations for the evaluation of left ventricular diastolic function by echocardiography. Eur J Echocardiogr. 2009;10:165–193 10.1093/ejechocard/jep007. 19270053

[23] Rudski LG, Lai WW, Afilalo J, Hua L, Handschumacher MD, Chandrasekaran K, et al Guidelines for the echocardiographic assessment of the right heart in adults: a report from the American Society of Echocardiography endorsed by the European Association of Echocardiography, a registered branch of the European Society of Cardiology, and the Canadian Society of Echocardiography. J Am Soc Echocardiogr. 2010;23:685–713; quiz 786–788 10.1016/j.echo.2010.05.010 20620859

[24] Hur E, Usta M, Toz H, Asci G, Wabel P, Kahvecioglu S, et al Effect of fluid management guided by bioimpedance spectroscopy on cardiovascular parameters in hemodialysis patients: a randomized controlled trial. Am J Kidney Dis. 2013;61:957–965 10.1053/j.ajkd.2012.12.017 23415416

[25] Bozbas SS, Akcay S, Altin C, Bozbas H, Karacaglar E, Kanyilmaz S, et al Pulmonary hypertension in patients with end-stage renal disease undergoing renal transplantation. Transplant Proc. 2009;41:2753–2756 10.1016/j.transproceed.2009.07.049 19765426

[26] Kawar B, Ellam T, Jackson C, Kiely DG. Pulmonary hypertension in renal disease: epidemiology, potential mechanisms and implications. Am J Nephrol. 2013;37:281–290 10.1159/000348804 23548763

[27] Abassi Z, Nakhoul F, Khankin E, Reisner SA, Yigla M. Pulmonary hypertension in chronic dialysis patients with arteriovenous fistula: pathogenesis and therapeutic prospective. Curr Opin Nephrol Hypertens. 2006;15:353–360 10.1097/01.mnh.0000232874.27846.37 16775448

[28] Etemadi J, Zolfaghari H, Firoozi R, Ardalan MR, Toufan M, Shoja MM, et al Unexplained pulmonary hypertension in peritoneal dialysis and hemodialysis patients. Rev Port Pneumol. 2012;18:10–14 10.1016/j.rppneu.2011.07.002 21920698

[29] Unal A, Duran M, Tasdemir K, Oymak S, Sipahioglu MH, Tokgoz B, et al Does arterio-venous fistula creation affects development of pulmonary hypertension in hemodialysis patients? Ren Fail. 2013;35:344–351 10.3109/0886022X.2012.760407 23356711

[30] Poulikakos D, Theti D, Pau V, Banerjee D, Jones D. The impact of arteriovenous fistula creation in pulmonary hypertension: measurement of pulmonary pressures by right heart catheterization in a patient with respiratory failure following arteriovenous fistula creation. Hemodial Int. 2012;16:553–555 10.1111/j.1542-4758.2012.00674.x 22360582

[31] Kim JS, Yang JW, Yoo JS, Choi SO, Han BG. Association between E/e´ ratio and fluid overload in patients with predialysis chronic kidney disease. PLoS One. 2017;12:e0184764 10.1371/journal.pone.0184764 28902883 PMC5597236

[32] Barberato SH, Mantilla DE, Misocami MA, Goncalves SM, Bignelli AT, Riella MC, et al Effect of preload reduction by hemodialysis on left atrial volume and echocardiographic Doppler parameters in patients with end-stage renal disease. Am J Cardiol. 2004;94:1208–1210 10.1016/j.amjcard.2004.07.100 15518627

[33] Mutlak D, Aronson D, Lessick J, Reisner SA, Dabbah S, Agmon Y. Functional tricuspid regurgitation in patients with pulmonary hypertension: is pulmonary artery pressure the only determinant of regurgitation severity? Chest. 2009;135:115–121 10.1378/chest.08-0277 18719061

[34] Galie N, Hoeper MM, Humbert M, Torbicki A, Vachiery JL, Barbera JA, et al Guidelines for the diagnosis and treatment of pulmonary hypertension: the Task Force for the Diagnosis and Treatment of Pulmonary Hypertension of the European Society of Cardiology (ESC) and the European Respiratory Society (ERS), endorsed by the International Society of Heart and Lung Transplantation (ISHLT). Eur Heart J. 2009;30:2493–2537 10.1093/eurheartj/ehp297 19713419

[35] Ramasubbu K, Deswal A, Herdejurgen C, Aguilar D, Frost AE. A prospective echocardiographic evaluation of pulmonary hypertension in chronic hemodialysis patients in the United States: prevalence and clinical significance. Int J Gen Med. 2010;3:279–286 10.2147/IJGM.S12946 21042428 PMC2962323

[36] Casas-Aparicio G, Castillo-Martinez L, Orea-Tejeda A, Abasta-Jimenez M, Keirns-Davies C, Rebollar-Gonzalez V. The effect of successful kidney transplantation on ventricular dysfunction and pulmonary hypertension. Transplant Proc. 2010;42:3524–3528 10.1016/j.transproceed.2010.06.026 21094809

[37] Bozbas SS, Kanyilmaz S, Akcay S, Bozbas H, Altin C, Karacaglar E, et al Renal transplant improves pulmonary hypertension in patients with end stage renal disease. Multidiscip Respir Med. 2011;6:155–160 10.1186/2049-6958-6-3-155 22959121 PMC3463070

